# Comparison of microsatellite distribution in the genomes of *Pteropus vampyrus* and *Miniopterus natalensis* (Chiroptera)

**DOI:** 10.1186/s12863-023-01108-7

**Published:** 2023-02-14

**Authors:** Weiwei Shao, Wei Cai, Fen Qiao, Zhihua Lin, Li Wei

**Affiliations:** College of Ecology, Lishui University, Lishui, 323000 Zhejiang People’s Republic of China

**Keywords:** Genome-wide identification, Microsatellite, Diversity, GO analysis, KEGG enrichment, Chiroptera

## Abstract

**Background:**

Microsatellites are a ubiquitous occurrence in prokaryotic and eukaryotic genomes. Microsatellites have become one of the most popular classes of genetic markers due to their high reproducibility, multi-allelic nature, co-dominant mode of inheritance, abundance and wide genome coverage. We characterised microsatellites in the genomes and genes of two bat species, *Pteropus vampyrus* and *Miniopterus natalensis*. This characterisation was used for gene ontology analysis and the Kyoto Encyclopedia of Genes and Genomes pathway enrichment of coding sequences (CDS).

**Results:**

Compared to *M. natalensis*, the genome size of *P. vampyrus* is larger and contains more microsatellites, but the total diversity of both species is similar. Mononucleotide and dinucleotide repeats were the most diverse in the genome of the two species. In each bat species, the microsatellite bias was obvious. The microsatellites with the largest number of repeat motifs in *P. vampyrus* from mononucleotide to hexanucleotide were (A)_n_, (AC)_n_, (CAA)_n_, (AAAC)_n_, (AACAA)_n_ and (AAACAA)_n_, with frequencies of 97.94%, 58.75%, 30.53%, 22.82%, 54.68% and 22.87%, respectively, while in *M. natalensis* were (A)_n_, (AC)_n_, (TAT)_n_, (TTTA)_n_, (AACAA)_n_ and (GAGAGG)_n_, with of 92.00%, 34.08%, 40.36%, 21.83%, 25.42% and 12.79%, respectively. In both species, the diversity of microsatellites was highest in intergenic regions, followed by intronic, untranslated and exonic regions and lowest in coding regions. Location analysis indicated that microsatellites were mainly concentrated at both ends of the genes. Microsatellites in the CDS are thus subject to higher selective pressure. In the GO analysis, two unique GO terms were found only in *P. vampyrus* and *M. natalensis*, respectively. In KEGG enriched pathway, the biosynthesis of other secondary metabolites and metabolism of other amino acids in metabolism pathways were present only in *M. natalensis*. The combined biological process, cellular components and molecular function ontology are reflected in the GO analysis and six functional enrichments in KEGG annotation, suggesting advantageous mutations during species evolution.

**Conclusions:**

Our study gives a comparative characterization of the genomes of microsatellites composition in the two bat species. And also allow further study on the effect of microsatellites on gene function as well as provide an insight into the molecular basis for species adaptation to new and changing environments.

## Background

Microsatellites or Simple-Sequence Repeats (SSRs) are tandemly repeated DNA sequences composed of mononucleotide, dinucleotide, trinucleotide, tetranucleotide, pentanucleotide and hexanucleotide units located throughout the prokaryotic [1] and eukaryotic genomes [2–4], in both non-coding and coding regions of DNA [5]. Moreover, retrotransposons may also be associated with microsatellites [6]. Furthermore, microsatellites have become one of the most popular classes of genetic markers due to their high reproducibility, multi-allelic nature, co-dominant mode of inheritance, abundance and wide genome coverage [3]. Despite their ubiquitous occurrence, microsatellite density and distribution vary significantly across genomes [7]. Moreover, high mutability at microsatellite loci contributes to genome evolution by creating genetic variation within a gene pool [8, 9]. Slipped-strand mispairing and subsequent error(s) during DNA replication, repair or recombination are the primary cause of this genetic variation [10, 11]. Strand slippage and unequal recombination results in the insertion or deletion of one to several repeated units. This high instability makes them attractive polymorphic molecular markers [12].

In recent years, in silico mining of microsatellite sequences from DNA-sequence databases has rapidly replaced the conventional methods for generating microsatellite markers from genomic libraries [13, 14]. Subsequently, several search tools are available for mining microsatellite repeats in assembled genome sequences, including Tandem Repeats Finder, Simple-Sequence Repeat Identification Tool, Tandem Repeats Occurrence Locator, SciRoko, MSDB and MIcroSAtellite (MISA) [3]. MISA is sophisticated and user-friendly microsatellite mining software [15]. Furthermore, MISA was performed for microsatellite mining in the genomes of *Anopheles sinensis* [16], *Epinephelus awoara* [17], *Boa constrictor* and *Protobothrops mucrosquamatus* [18], *Nanorana parkeri* and *Xenopus laevis* [19]. These investigations indicate that microsatellites are found less frequently in protein-coding sequences than in intronic and intergenic regions [18]. Microsatellites in coding regions are more diverse than those in non-coding regions due to higher coding density [20]. The microsatellite length expansion may affect gene regulation, transcription and protein function of coding sequences (CDS), particularly for trinucleotide repeats, which are associated with human diseases [21], such as Huntington and Machado-Joseph disease [22], neurological disease [23] and colorectal cancer [24]. Microsatellite distribution characteristics and functions may vary among genomes [25]. Therefore, whole genome sequencing encourages the development of microsatellite markers derived from the database [3, 26].

In the present study, we investigated the Chiroptera genomes of the large flying fox (*Pteropus vampyrus*) and Natal long-fingered bat (*Miniopterus natalensis*) that have been reported in the open databases. *P. vampyrus* is the largest of any bat species belonging to Yinpterochiroptera that cannot vocalise echolocation calls [27], whereas *M. natalensis* is a representative species of Yangochiroptera that can produce modulated frequency (FM) echolocation calls [28]. Furthermore, we analysed the characteristics and functional annotation of microsatellites at the genomic level of the two bat species. These findings should contribute to our understanding of the bat genome and facilitates subsequent screening and development of large numbers of high-quality microsatellite markers.

## Methods

The *P. vampyrus* genome assembly was downloaded from the National Center for Biotechnology Information (NCBI) under BioProject accession PRJNA20325, with annotation files downloaded from https://ftp.ncbi.nlm.nih.gov/genomes/all/GCF/000/151/845/GCF_000151845.1_Pvam_2.0/, including CDS sequences. Similarly, the genome assembly of *M. natalensis* was downloaded from NCBI under BioProject accession PRJNA283550, with annotation files downloaded from https://ftp.ncbi.nlm.nih.gov/genomes/all/GCF/001/595/765/GCF_001595765.1_Mnat.v1/, including CDS sequences. Microsatellites in the genome and CDS were identified using MISA identification tool software, which has been used for microsatellite analysis of several species, including *Nanorana parkeri* (high Himalaya frog), *Xenopus laevis* (African clawed frog) [19], *Boa constrictor (red-tailed boa)* and *Protobothrops mucrosquamatus* (brown-spotted pit viper) [18]. Def in the misa.ini file was set as 1–12, 2–6, 3–5, 4–5, 5–4 and 6–4 to restrict the detection criteria for perfect SSR of 1–6 bp with minimum repeat numbers of 12, 6, 5, 5, 4 and 4 for mononucleotide, dinucleotide, trinucleotide, tetranucleotide, pentanucleotide and hexanucleotide microsatellites, respectively [29, 30]. Further, when the distance between two microsatellites was shorter than 100 bp, they were considered single-compound microsatellites [31]. Moreover, repeats with unit patterns being circular permutations and/or reverse complements were considered as one type [32, 33], such as the AAG contains CTT, AGA, TCT, GAA, and TTC or GCGT contains ACGC, CGTG, CACG, GTGC, GCAC, TGCG, and CGCA in different reading frames or on the complementary strand.

Furthermore, the frequency and diversity of SSRs in each bat genome were calculated. The frequency was determined as the percentage of the total number of SSRs per megabase (Mb) of the genome sequence. The diversity of microsatellites, which is the SSR number per Mb of the sequence analysed, was calculated using the methods reported in the literature by Fujimori et al. [31], Qian et al. [34], Nie et al. [18] and Wei et al. [19]. The relative positions of the exon, intron, gene and intergene regions were extracted from the annotation files via custom Python scripts to explore the distribution of microsatellites in the genomes of *P. vampyrus* and *M. natalensis* [16]. The microsatellites on different regions of the genes were then located. The genes were divided into 13 elements containing 500 bp upstream, the first exon/intron, second exon/intron, middle left exon, middle intron, middle right exon, last second intron, last second exon, last intron, last exon and 500 bp downstream [18, 19]. Further, to avoid overlap in measurements, only genes with more than six exons and five introns were considered [31]. The relative position (from P0.1 to P1.0) of a microsatellite in a certain type of element is the distance from the microsatellite to the left end of the element divided by the distance between the length of the element and the length of the microsatellite [19].

CDS with microsatellites were aligned against NCBI non-redundant and SWISS-PROT protein databases (http://www.uniprot.org) and the Kyoto Encyclopedia of Genes and Genomes (KEGG) database (http://www.genome.jp/kegg), using BLASTx with an E-value threshold of 1e^−5^ [35]. Protein functional annotations were then obtained according to the best alignment results. The Blast2GO software was used to analyse the gene ontology (GO) annotation of genes [36], and WEGO software was employed to investigate the functional classification of genes such as biological processes, cellular components and molecular function [37].

## Results

### Microsatellite frequency and distribution in the genomes of the two species

Table 1 shows the results of the microsatellite analysis. A total of 512,647 SSRs were found in the genome assembly of approximately 2.20 Gb for *P. vampyrus,* and a total of 448,674 SSRs were found in the genome assembly of approximately 1.80 Gb for *M. natalensis*. The SSR content of the genome between species was similar, with 0.46% in *P. vampyrus* and 0.47% in *M. natalensis*. Additionally, the total microsatellite diversity between species was similar, i.e., 233.20 SSRs/Mb in *P. vampyrus* and 248.83 SSRs/Mb in *M. natalensis*. The mononucleotide motifs were the most abundant category, followed by dinucleotide and tetranucleotide motifs for *P. vampyrus*. Whereas in *M. natalensis*, dinucleotide repeats were the most diversified category, followed by mononucleotide and tetranucleotide repeats (Table 1). The most diverse SSR types from mononucleotide to hexanucleotide motifs in *the P. vampyrus* genome were (A)_n_, (AC)_n_, (CAA)_n_, (AAAC)_n_, (AACAA)_n_ and (AAACAA)_n_ and in *M. natalensis* were (A)_n_, (AC)_n_, (TAT)_n_, (TTTA)_n_, (AACAA)_n_ and (GAGAGG)_n_. Moreover, similarities between species were noted in dinucleotide (TA)_n_, (GT)_n_, (GA)_n_ and (GC)_n_, trinucleotides (CAT)_n_, tetranucleotides (ATAG)_n_ and (CATT)_n_, in pentanucleotide (AACAA)_n_, (TTATT)_n_ and (TTTCT)_n_ and in hexanucleotide (CTGTCT)_n_. Table 2 shows the concentration of differences in trinucleotide, tetranucleotide, pentanucleotide and hexanucleotide types (Table 2).

**Table 1:** Distribution of microsatellites in the genomes of *Pteropus vampyrus* and *Miniopterus natalensis*

Motif length	*Pteropus vampyrus*				*Miniopterus natalensis*			
	Numbers of microsatellites	Length(bp)	Abundance (SSRs/Mb)	Frequency (%)	Numbers of microsatellites	Length(bp)	Abundance (SSRs /Mb)	Frequency (%)
Mononucleotide	246,947	3,647,964	112.34	48.17	144,835	2,174,691	80.33	32.28
Dinucleotide	163,249	3,649,342	74.26	31.84	235,344	4,030,076	130.52	52.45
Trinucleotide	36,521	750,138	16.61	7.12	20,959	386,283	11.62	4.67
Tetranucleotide	43,966	1,409,268	20.00	8.58	32,493	1,259,172	18.02	7.24
Pentanucleotide	15,137	382,635	6.89	2.95	10,320	367,900	5.72	2.30
Hexanucleotide	6827	199,332	3.11	1.33	4723	188,970	2.62	1.05
Total	512,647	10,038,679	233.20	100.00	448,674	8,407,092	248.83	100.00
Whole genome length/bp	2,198,284,804				1,803,099,001			
SSR content in the genome	0.46%				0.47%

**Table 2:** The most frequent microsatellite motifs found in the genomes of *Pteropus vampyrus* and *Miniopterus natalensis*

Motif length	*Pteropus vampyrus*			*Miniopterus natalensis*		
	Repeat unit	Microsatellites	Frequency (%)	Repeat unit	Microsatellites	Frequency (%)
Mononucleotide	A	241,850	97.94	A	133,249	92.00
	G	5097	2.06	G	11,586	8.00
Dinucleotide	AC	95,909	58.75	AC	80,208	34.08
	CT	37,060	22.70	CT	126,869	53.91
	GC	1394	0.85	GC	467	0.20
	TA	28,886	17.69	TA	27,800	11.81
Trinucleotide	CAA	11,151	30.53	TAT	8458	40.36
	TAT	9997	27.37	CAA	3341	15.94
	CAT	4202	11.51	CAT	2508	11.97
	GAG	2974	8.14	ACC	2380	11.36
Tetranucleotide	AAAC	10,035	22.82	TTTA	7092	21.83
	ATAG	6429	14.62	ATAG	5488	16.89
	CATT	5268	11.98	CATT	3802	11.70
	TTTA	4488	10.21	CCTT	3721	11.45
Pentanucleotide	AACAA	8277	54.68	AACAA	2623	25.42
	TTATT	2174	14.36	TTATT	2515	24.37
	TTTCT	851	5.62	TTTCT	621	6.02
	CCACC	295	1.95	AGGGA	606	5.87
Hexanucleotide	AAACAA	1561	22.87	GAGAGG	604	12.79
	GGGTTA	1282	18.78	TATCTA	271	5.74
	CTGTCT	442	6.47	CTGTCT	261	5.53
	TATCTA	414	6.06	GGGTTA	215	4.55

The 15 most diverse microsatellite repeats in the *P. vampyrus* genome were (A)_n_, (AC)_n_, (CT)_n_, (TA)_n_, (CAA)_n_, (AAAC)_n_, (TAT)_n_, (AACAA)_n_, (ATAG)_n_, (CATT)_n_, (G)_n_, (TTTA)_n_, (CCTT)_n_, (CAT)_n_ and (GAG)_n_ comprising of 92.84% of all microsatellites identified. Similarly, the 15 most diverse microsatellite motifs in *M. natalensis* were (A)_n_, (CT)_n_, (AC)_n_, (TA)_n_, (G)_n_, (TAT)_n_, (TTTA)_n_, (ATAG)_n_, (CATT)_n_, (CCTT)_n_, (CAA)_n_, (TGGA)_n_, (AACAA)_n_, (AAAC)_n_ and (TTATT)_n_ comprising of 94.10% of all microsatellites identified.

**Fig. 1:**
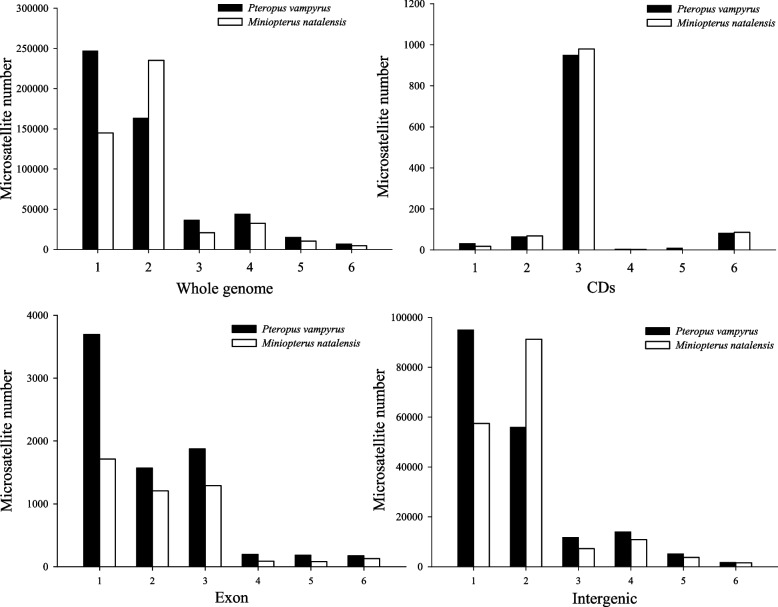
Distribution of microsatellite types in different genomic regions of *Pteropus vampyrus* and *Miniopterus natalensis*. 1–6 indicated mononucleotide, dinucleotide, trinucleotide, tetranucleotide, pentanucleotide, and hexanucleotide unit length, respectively

Table 3 displays the distributions of microsatellites in the genomes of *P. vampyrus* and *M. natalensis*. Intergenic regions had the most numbers of microsatellites, and CDS exhibited a few in both species. The number of microsatellites in the intergenic, intron, exon and untranslated regions of *P. vampyrus* was greater than that in *M. natalensis*; however, the diversity of microsatellites in intron regions of *P. vampyrus* was less than that in *M. natalensis.* The numbers and diversity of microsatellites in CDS in *M. natalensis* were larger than those in *P. vampyrus*. Further, microsatellites in the CDS were found to be less diverse than those in other regions. Figure 1 illustrates the frequency of different microsatellite types in different genomic regions. In both species, trinucleotides were the most diverse microsatellite type in CDS, with 83.11% and 84.70% in *P. vampyrus* and *M. natalensis*, respectively. The numbers of mononucleotide, dinucleotide, trinucleotide, tetranucleotide, pentanucleotide and hexanucleotide in the exons of *P. vampyrus* were much greater than that of *M. natalensis*. The distribution of SSRs in intergenic regions was similar to the distribution in whole genomes, with the most diversity among mononucleotides and dinucleotides.

**Table 3:** The number and diversity (microsatellites/Mb) of microsatellites in different genomic regions of *Pteropus vampyrus* and *Miniopterus natalensis*

Species	Gene				Intergenic
	CDs	Untranslated	Exon	Intron	
*Pteropus vampyrus*	1143(355.55)	4710(1537.15)	7702(1226.66)	183,514(2244.51)	402,059(3050.79)
*Miniopterus natalensis*	1157(371.89)	2953(1323.03)	4503(842.76)	171,977(2436.11)	292,798(2805.33)

### Location analysis of microsatellites in genes

**Fig. 2:**
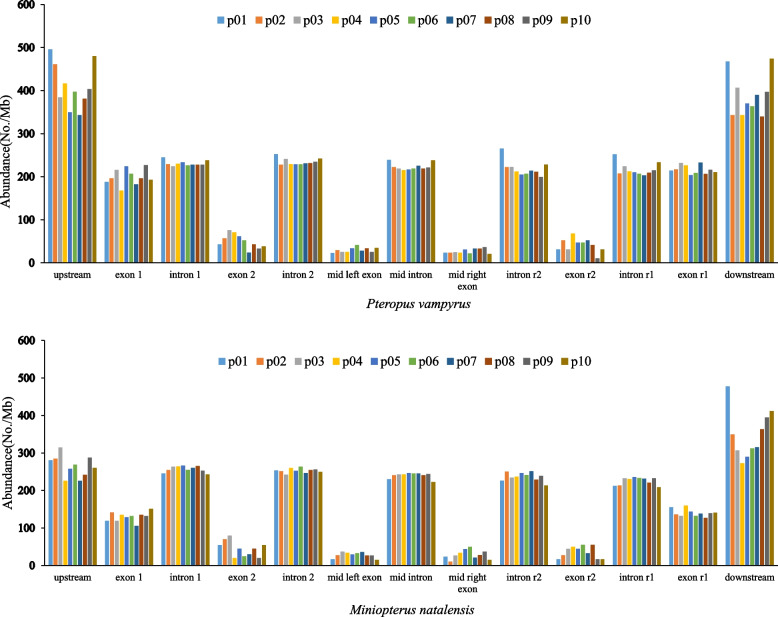
Microsatellite abundance in gene regions and their upstream and downstream regions of *Pteropus vampyrus* and *Miniopterus natalensis*

All microsatellites in exons or introns were compared with 979 and 1010 genes, with more than six exons and five introns in *P. vampyrus* and *M. natalensis*, respectively. Microsatellite-enriched regions were upstream and downstream of genes in both *P. vampyrus* and *M. natalensis* genomes, with the numbers of microsatellites in exons, gradually decreasing from the first exon toward the last second exon and increasing toward the last exon (Fig. 2). In each bat species, microsatellite diversity in upstream and downstream regions was similar. Likewise, microsatellite diversity in various introns was also similar (Fig. 2).

### Functional analysis of CDS with microsatellites for two species

**Fig. 3:**
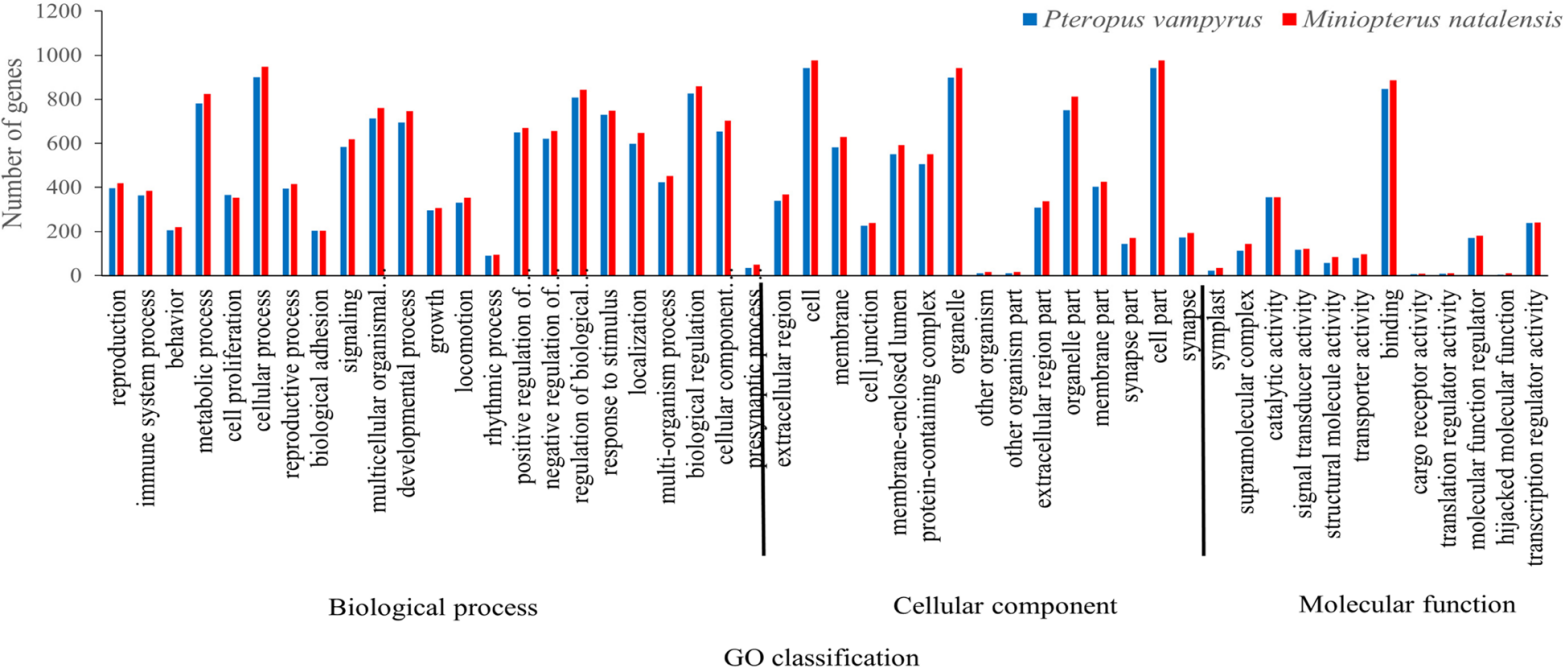
GO classifications of coding sequencing (CDS) with microsatellites in the genomes of *Pteropus vampyrus* and *Miniopterus natalensis*

In genomes of *P. vampyrus* and *M. natalensis*, 1019 and 1043 CDS with SSR, respectively, were imported into GO analysis based on sequence alignment. All these CDS were assigned to 20572 (*P. vampyrus*) and 21816 (*M. natalensis*) GO in terms of their known functions. Figure 3 shows the number of CDS with SSRs assigned to each subcategory. Further, 50 pairs were represented in both species of these GO functional classifications. Carbon utilisation (GO: 0015976) and biological phase (GO: 0044848) in the biological process ontology were only present in *P. vampyrus*, while the virion (GO: 0019012) and virion part (GO: 0044423) in cellular component ontology were present only in *M. natalensis*. Furthermore, comparing the function distribution between the two species, cellular process (GO: 0009987) in biological process ontology was most frequent. Cell (GO: 0005623) and cell part (GO: 0044464) were the top two terms in the cellular component ontology. In the molecular function ontology, binding (GO: 0005488) was prominent.

**Fig. 4:**
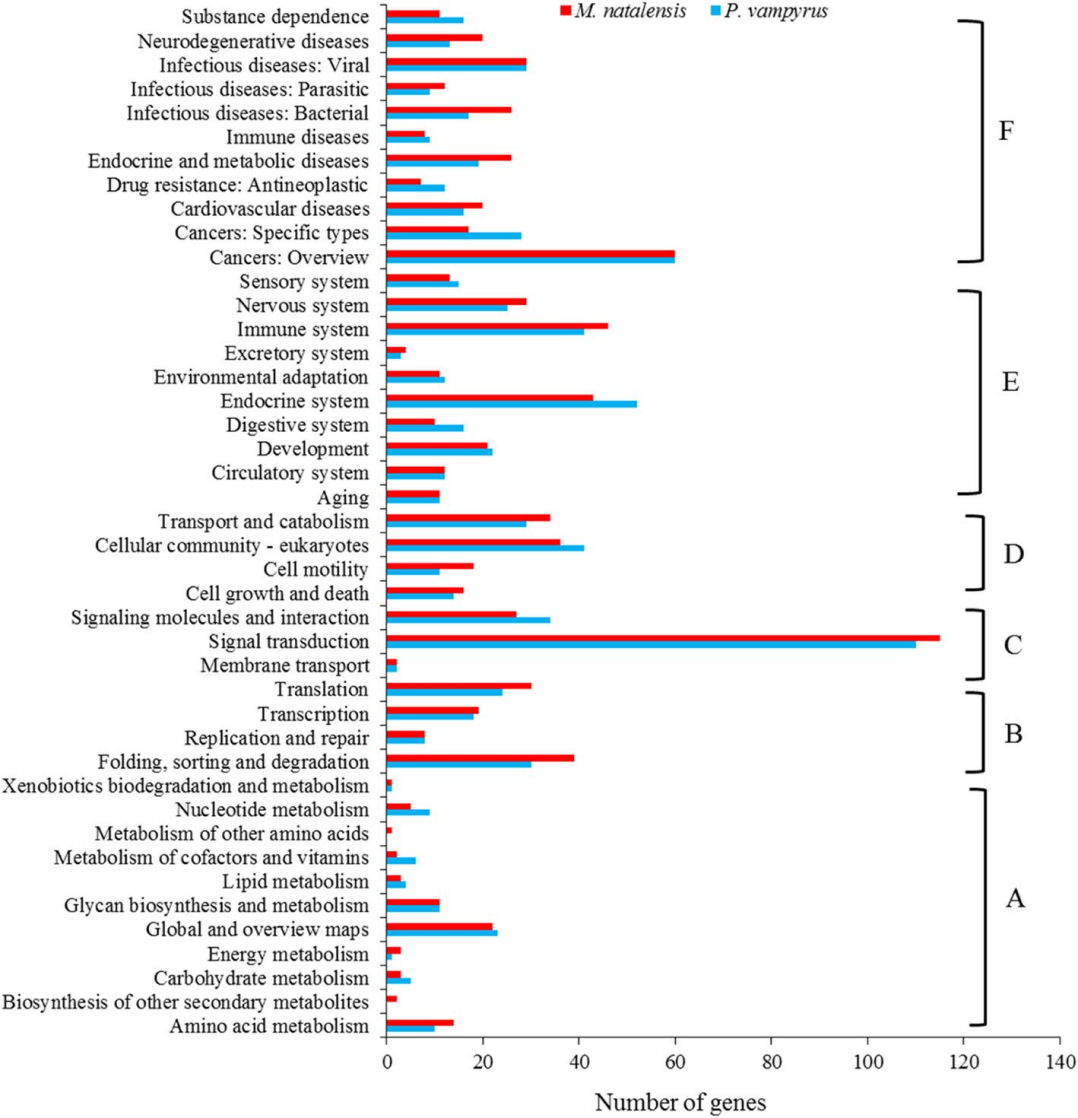
KEGG enrichment of microsatellites with CDS in *Pteropus vampyrus* and *Miniopterus natalensis*: (**A**) Metabolism, (**B**) Environmental information processing, (**C**) Genetic information processing, (**D**) Cell process, (**E**) Organismal systems and (**F**) Human diseases and drug development

CDS were assigned to 828 for *P. vampyrus* and 847 for *M. natalensis* in terms of known functions for KEGG annotation. Figure 4 shows these KO functional classifications indicating that 41 and 43 pathways were enriched in *P. vampyrus* and *M. natalensis*, respectively. All the enrichment pathways were divided into six functional classification categories, i.e., metabolism, environmental information processing, genetic information processing, cell process, organismal systems and human diseases and drug development (Fig. 4). The biosynthesis of other secondary metabolites and metabolism of other amino acids in metabolism pathways were present only in *M. natalensis*. Among these pathways, the signal transduction pathway was the most enriched, with 110 genes in *P. vampyrus* and 115 genes for *M. natalensis*.

## Discussion

Genome-wide identification of SSR markers have been successfully performed in various animals [38]. To our best knowledge, the present study is the comprehensive report on the characterization of microsatellites in bat species of *P. vampyrus* and *M. natalensis*. Genome size, total number of SSR and total length of SSR identified in *P. vampyrus* were all larger than those in *M. natalensis* (Table 1). These differences in genomes of the two species may be caused by their genome size, assembly quality, the number of positions of the unknown base and specificity of species [3, 39]. This phenomenon has been reported in other species, such as *B. constrictor* and *P. mucrosquamatus* [18], *Tetranychus urticae* and *Ixodes scapularis* [40] and *Phytophthora* [41]. However, microsatellite content in the genomes of *P. vampyrus* and *M. natalensis* was similar, accounting for 0.46% and 0.47%, respectively. This result is consistent with other bat species *Rhinolophus ferrumequinum* (0.58%, unpublished data) and *Hipposideros armiger* (0.50%, unpublished data), as well as previous studies in other mammals, such as giant panda (*Ailuropoda melanoleuca*, 0.64%), the polar bear (*Ursus maritimus*, 0.79%) [42] and forest musk deer (*Moschus berezovskii*, 0.42%) [43]. Total SSR diversity in the genomes of *P. vampyrus* and *M. natalensis* are 233.20 SSRs/Mb and 248.83 SSRs/Mb, respectively, which were lower in comparison to the diversity of *R. ferrumequinum* with 263.65 SSRs/Mb (unpublished data) but higher compared to the diversity of *H. armiger* (222.61 SSRs/Mb (unpublished data). This indicates that the genomic size and quality of sequencing have a great influence on the identification of microsatellites [18].

The sequence proportions of six SSR types in *P. vampyrus* and *M. natalensis* genomes are different, as are the four most diverse microsatellite types (Table 2). This result has also been reported in patterns of genomic SSRs of *N. parkeri* and *X. laevis* [19], *B. constrictor* and *P. mucrosquamatus* [18], *C. exilicauda* and *M. martensii* [44]. However, genomes of *Eucryptorrhynchus brandti* and *E. scrobiculatus* exhibit similarities in the six SSR types [45] suggesting that the differences and similarities in microsatellite composition in the genome can reflect the relationship among species to some extent [46]. Frequency and abundance analysis of various motif repeats in *P. vampyrus* genome revealed that mononucleotide repeats were the dominant type of SSRs (Table 1). These results are in agreement with previous studies in other eukaryotic organisms. For example, mononucleotide was the dominant SSR types in *Lophophorus lhuysii* [47], *M. berezovskii* [43] and *Macaca fascicularis* [48]. On the contrary, dinucleotide was the dominant SSR types in the genome of *M. natalensis*, which is in agreement with other species of *N. parkeri* and *X. laevis* [19], *Rhodeus sinensis* [49] and *Eriocheir sinensis* [50]. Dinucleotides were the dominant types because of their higher mutation rates [37]. For example, dinucleotides in human nonpathogenic SSR loci have mutation rates of 1.5–2 times higher than tetranucleotides [51].

In comparisons with *P. vampyrus* and *M. natalensis*, differences in both frequency and diversity of SSRs in CDS were minor, whereas those in exon, intron, untranslated and intergenic regions were significant (Table 3). Furthermore, the diversity of microsatellites in untranslated regions was greater than those in CDS regions, indicating that microsatellites aggregate in untranslated regions, presumably influencing gene transcriptional activity [52]. Coding regions are generally conservative among different species and are subject to high-selective pressure [53]. In this study, trinucleotide SSRs in the CDS were the most diverse SSR types in both bat species. Further, the diversity of trinucleotide SSRs in the CDS of the *M. natalensis* genome is greater than that in the *P. vampyrus*, possibly due to the faster rate of evolution of *M. natalensis*. This phenomenon could be explained by an increase in trinucleotide repetitions in coding regions, which can increase trait diversity and facilitate adaptive changes in response to environmental alterations [54]. Therefore, the characteristics of microsatellite repeats in the genomes of various species could be reflected in their different dominants [3].

*P. vampyrus* and *M. natalensis* had different SSR locations in genes (Fig. 2). SSRs in the upstream and downstream regions of both species were similar, with the highest diversity. Instead, SSR diversity in upstream and downstream regions of *P. vampyrus* was greater than in *M. natalensis,* predicting the underlying reason for the larger genome size of *P. vampyrus*. In each species, SSR diversity in exons showed a “U” shape that gradually decreased from the first exon toward the last second exon and then increased toward the last exon. This phenomenon is consistent with *C. exilicauda* and *M. martensii* reported by Wang et al. [44], and *B. constrictor* and *P. mucrosquamatus* reported by Nie et al. [18], respectively. SSR diversity in various introns was similar in each of the two species. Therefore, comparisons of SSR diversity in gene regions between the two species revealed that different numbers and diversity of SSR in genes may facilitate adaptation to evolutionary history. *P. vampyrus* is a fruit-eating bat that usually roosts in trees and has non-echolocation calls, whereas *M. natalensis* is an insectivorous bat with echolocation calls that primarily live in caves and mines that are used for hibernation and reproduction [27].

For functional annotation of coding genes, GO analysis found two (GO: 0015976 and GO: 0044848) for *P. vampyrus* and two (GO: 0019012; GO: 0044423) unique GO terms for *M. natalensis*, respectively, indicating a significant difference in the genomes between species. Moreover, many CDS with SSRs are associated with environmental interactions, such as metabolic processes (GO: 0008152), cellular processes (GO: 0009987), signalling (GO: 0023052) and response to stimulus (GO:0050896), which may be related to the different adaptability to the environment of the two bats. This pattern is also reported in a study of *N. parkeri* and *X. laevis* [19]. In KEGG annotation, 41 and 43 pathways were enriched in *P. vampyrus* and *M. natalensis*, respectively. We found that two (Biosynthesis of other secondary metabolites and metabolism of other amino acids) unique metabolism pathways were presented only in *M. natalensis*, which may further indicate some significantly different functions in the genes between species. In both species, genetic information processing has the fewest pathways, with only 3 pathways containing 146 genes in *P. vampyrus* and 144 genes in *M. natalensis*. Human diseases and drug development have the most pathways, with 11 pathways containing 228 genes in *P. vampyrus* and with 9 pathways containing 236 genes in *M. natalensis*, respectively, suggesting that bats are one of the most important natural hosts of mammalian viruses [55]. There are 28 families of viruses found in bats [56]. A recent study showed that the homology of the outbreak of the new coronavirus (Covid-19) in late 2019 is 79% compared to SARS-CoV at the genome-wide level and up to 89% compared to SARRr ZC45 sampled from a *Rhinolophus* bat in Zhejiang, China [57]. As different coronaviruses recombine to produce new viruses, SSRs in the genes of bats may evolve in adaptive changes to internal alterations and, consequently, remain fit in zoonosis [58–60].

## Conclusions

As summarised above, characteristics of microsatellites at the genomic level of *P. vampyrus* and *M. natalensis* were analysed and compared in this study. Further, the classification and functional evolution of genes with SSRs in these two bat species should continue; results will contribute to a further understanding of the evolutionary history of other Chiroptera species.

## Data Availability

The datasets generated and/or analysed during the current study are available in the National Center for Biotechnology Information (NCBI) repository. The *Pteropus vampyrus* genome assembly was downloaded from BioProject accession PRJNA20325, with annotation files downloaded from https://ftp.ncbi.nlm.nih.gov/genomes/all/GCF/000/151/845/GCF_000151845.1_Pvam_2.0/, including CDS sequences. Similarly, the genome assembly of *Miniopterus natalensis* was downloaded from BioProject accession PRJNA283550, with annotation files downloaded from https://ftp.ncbi.nlm.nih.gov/genomes/all/GCF/001/595/765/GCF_001595765.1_Mnat.v1/, including CDS sequences.
